# Supplementary material for: Domestic ecologies of gender: Women, material life, and eco-folklore in Saudi proverbs
Source: PLoS One. 2026 Jun 10;21(6):e0348777. doi: 10.1371/journal.pone.0348777 (PMC13252737; doi:10.1371/journal.pone.0348777)
Supplement: Attachment — Submitted filename: PONE-D-25-68737_reviewer.pdf [file pone.0348777.s001.pdf]

PLOS One

Domestic Ecologies of Gender: Women, Material Life, and Eco-Folklore in Saudi Proverbs

--Manuscript Draft--

**Manuscript Number:**	PONE-D-25-68737
**Article Type:**	Research Article
**Full Title:**	Domestic Ecologies of Gender: Women, Material Life, and Eco-Folklore in Saudi Proverbs
**Short Title:**	Domestic Ecologies of Gender: Women, Material Life, and Eco-Folklore in Saudi Proverbs
**Corresponding Author:**	Ali M Alshhre, Ph.D.<br>King Khalid University College of Languages and Translation<br>Abha, SAUDI ARABIA
**Keywords:**	Saudi proverbs; women in folklore; eco-folklore; material ecocriticism; feminist discourse analysis; domestic ecology; vernacular environmental knowledge; gender ideology; Gulf cultural studies
**Abstract:**	This article advances an ecocritical reading of women-focused Saudi proverbs as a distinctive form of eco-folklore, where gendered norms are naturalized through domestic material and environmental imaginaries. Analyzing a corpus of 60 proverbs from the Saudi National Encyclopedia's regional archives, the study integrates feminist critical discourse analysis with material ecocriticism to reveal how proverbial discourse mobilizes everyday objects and processes bread and heat as emblems of maternal care, cooking vessels as symbols of household responsibility, pests like flies and scorpions as metaphors for moral contamination, and adornments such as kohl and henna as mechanisms of reputational surveillance—to position women as the pivotal agents in a gendered domestic ecosystem. The findings highlight an ambivalent moral economy: proverbs ostensibly praising women for motherhood, marital devotion, and "building" the home often reinforce unequal labor and accountability, while cautionary sayings discipline female mobility, visibility, and autonomy through idioms of scarcity, risk, and depletion. By framing Saudi paremiology as vernacular environmental reasoning embedded in gender ideology, this study contributes to Gulf cultural studies, folklore scholarship, and ecofeminist approaches to vernacular knowledge, demonstrating how proverbs function as micro-archives of everyday ecological governance.
**Order of Authors:**	Ali M Alshhre, Ph.D.
**Additional Information:**	
**Question**	**Response**
**Financial Disclosure**<br><br>Enter a financial disclosure statement that describes the sources of funding for the work included in this submission. Review the submission guidelines for detailed requirements. View published research articles from *PLOS ONE* for specific examples.<br><br>This statement is required for submission and **will appear in the published article** if the submission is accepted. Please make sure it is accurate.	The author(s) received no specific funding for this work.

**Funded studies**<br>Enter a statement with the following details:<br>• Initials of the authors who received each award<br>• Grant numbers awarded to each author<br>• The full name of each funder<br>• URL of each funder website<br>• Did the sponsors or funders play any role in the study design, data collection and analysis, decision to publish, or preparation of the manuscript?<br><br>Did you receive funding for this work?	
**Competing Interests**<br><br>Use the instructions below to enter a competing interest statement for this submission. On behalf of all authors, disclose any competing interests that could be perceived to bias this work—acknowledging all financial support and any other relevant financial or non-financial competing interests.<br><br>This statement is required for submission and **will appear in the published article** if the submission is accepted. Please make sure it is accurate and that any funding sources listed in your Funding Information later in the submission form are also declared in your Financial Disclosure statement.<br><br>View published research articles from *PLOS ONE* for specific examples.	The author has declared that no competing interests exist.

**NO authors have competing interests**<br><br>Enter: *The authors have declared that no competing interests exist.*<br><br>**Authors with competing interests**<br><br>Enter competing interest details beginning with this statement:<br><br>*I have read the journal's policy and the authors of this manuscript have the following competing interests: [insert competing interests here]*<br><br>* typeset	
**Ethics Statement**<br><br>Enter an ethics statement for this submission. This statement is required if the study involved:<br><br>• Human participants<br>• Human specimens or tissue<br>• Vertebrate animals or cephalopods<br>• Vertebrate embryos or tissues<br>• Field research<br><br>Write "N/A" if the submission does not require an ethics statement.<br><br>General guidance is provided below. Consult the submission guidelines for detailed instructions. **Make sure that all information entered here is included in the Methods section of the manuscript.**	"N/A"

**Format for specific study types**<br><br>**Human Subject Research (involving human participants and/or tissue)**<br>• Give the name of the institutional review board or ethics committee that approved the study<br>• Include the approval number and/or a statement indicating approval of this research<br>• Indicate the form of consent obtained (written/oral) or the reason that consent was not obtained (e.g. the data were analyzed anonymously)<br><br>**Animal Research (involving vertebrate animals, embryos or tissues)**<br>• Provide the name of the Institutional Animal Care and Use Committee (IACUC) or other relevant ethics board that reviewed the study protocol, and indicate whether they approved this research or granted a formal waiver of ethical approval<br>• Include an approval number if one was obtained<br>• If the study involved *non-human primates*, add *additional details* about animal welfare and steps taken to ameliorate suffering<br>• If anesthesia, euthanasia, or any kind of animal sacrifice is part of the study, include briefly which substances and/or methods were applied<br><br>**Field Research**<br><br>Include the following details if this study involves the collection of plant, animal, or other materials from a natural setting:<br>• Field permit number<br>• Name of the institution or relevant body that granted permission	
**Data Availability**<br><br>Authors are required to make all data underlying the findings described fully available, without restriction, and from the time of publication. PLOS allows rare exceptions to address legal and ethical concerns. See the PLOS Data Policy and FAQ for detailed information.	Yes - all data are fully available without restriction

A Data Availability Statement describing where the data can be found is required at submission. Your answers to this question constitute the Data Availability Statement and **will be published in the article**, if accepted.<br><br>**Important:** Stating 'data available on request from the author' is not sufficient. If your data are only available upon request, select 'No' for the first question and explain your exceptional situation in the text box.<br><br>Do the authors confirm that all data underlying the findings described in their manuscript are fully available without restriction?	
**Describe where the data may be found in full sentences. If you are copying our sample text, replace any instances of XXX with the appropriate details.**<br><br>• If the data are **held or will be held in a public repository**, include URLs, accession numbers or DOIs. If this information will only be available after acceptance, indicate this by ticking the box below. For example: *All XXX files are available from the XXX database (accession number(s) XXX, XXX.)*.<br>• If the data are all contained **within the manuscript and/or Supporting Information files**, enter the following: *All relevant data are within the manuscript and its Supporting Information files.*<br>• If neither of these applies but you are able to provide **details of access elsewhere**, with or without limitations, please do so. For example:<br><br>*Data cannot be shared publicly because of [XXX]. Data are available from the XXX Institutional Data Access / Ethics Committee (contact via XXX) for researchers who meet the criteria for access to confidential data.*<br><br>*The data underlying the results presented in the study are available from (include the name of the third party*	proverbs used for analysis are in the body of the manuscript

and contact information or URL).<br>• This text is appropriate if the data are owned by a third party and authors do not have permission to share the data.<br><br>* typeset	
Additional data availability information:	Tick here if the URLs/accession numbers/DOIs will be available only after acceptance of the manuscript for publication so that we can ensure their inclusion before publication.

Powered by Editorial Manager® and ProduXion Manager® from Aries Systems Corporation

Manuscript Click here to access/download;Manuscript;Manuscript author details.docx

Domestic Ecologies of Gender: Women, Material Life, and Eco-Folklore in Saudi Proverbs

Ali M. Alshhre
Associate Professor of Comparative Literature
English Department, College of Languages and Translation
King Khalid University, Abha, Saudi Arabia
ashehre@kku.edu.sa
https://orcid.org/0000-0003-4907-8979

Abstract

This article advances an ecocritical reading of women-focused Saudi proverbs as a distinctive form of eco-folklore, where gendered norms are naturalized through domestic material and environmental imaginaries. Analyzing a corpus of 60 proverbs from the Saudi National Encyclopedia's regional archives, the study integrates feminist critical discourse analysis with material ecocriticism to reveal how proverbial discourse mobilizes everyday objects and processes bread and heat as emblems of maternal care, cooking vessels as symbols of household responsibility, pests like flies and scorpions as metaphors for moral contamination, and adornments such as kohl and henna as mechanisms of reputational surveillance—to position women as the pivotal agents in a gendered domestic ecosystem. The findings highlight an ambivalent moral economy: proverbs ostensibly praising women for motherhood, marital devotion, and "building" the home often reinforce unequal labor and accountability, while cautionary sayings discipline female mobility, visibility, and autonomy through idioms of scarcity, risk, and depletion. By framing Saudi paremiology as vernacular environmental reasoning embedded in gender ideology, this study contributes to Gulf cultural studies, folklore scholarship, and ecofeminist approaches to vernacular knowledge, demonstrating how proverbs function as micro-archives of everyday ecological governance.

***Keywords**: Saudi proverbs; women in folklore; eco-folklore; material ecocriticism; feminist discourse analysis; domestic ecology; vernacular environmental knowledge; gender ideology; Gulf cultural studies*

1. Introduction

In contemporary Saudi Arabia, the sweeping reforms of Vision 2030 have reconfigured the social landscape, propelling women's labor force participation to 34.5% as of Q2 2025, dismantling longstanding mobility restrictions, and positioning gender equality as a cornerstone of economic diversification. Yet even as official discourse celebrates women as central to national progress, traditional cultural forms such as proverbs continue to circulate with enduring rhetorical force (1,2). These sayings, preserved in heritage collections and repeated in everyday life, often anchor gender norms in material and ecological imaginaries that appear timeless and self-evident. Their portability, anonymity, repeatability, and authority render them powerful disciplinary technologies, especially when the subject of judgment is "woman." In this way, proverbs intersect uneasily with modernization narratives: while state slogans proclaim empowerment, proverbial wisdom can reinscribe caution, reputational surveillance, and moral risk into daily interactions. The persistence of these forms raises critical questions about whether they quietly reinforce ambivalence toward women's expanded roles, even as the nation publicly embraces transformation.

Proverbs are among the most portable cultural forms in vernacular life: they circulate without authorship, travel across households and generations, and return in moments of decision as if they were self-evident verdicts rather than contested interpretations. In proverb studies, this portability is not merely a stylistic feature; it is the mechanism through which a community's judgments become socially reusable, allowing a short utterance to do the work of explanation, warning, or legitimation in a single breath (1, 2). When the subject of that judgment is "woman," the proverb often becomes a disciplinary technology precisely because it is small, repeatable, and difficult to argue with in everyday settings: it arrives already wrapped in the authority of "what people say," and thus can naturalize gendered expectations as common sense rather than as ideology (3, 4). Yet the authority of women-focused proverbs is also material and ecological. These sayings rarely build their claims through abstract moral principles alone; they reason through objects, substances, animals, and household processes, bread, pots, kohl, henna, flies, scorpions, exhaustion, and the domestic spaces where labor and reputation are produced. This article argues that women-focused Saudi proverbs constitute a form of eco-folklore: a vernacular archive in which gender norms are articulated through environmental and material imaginaries, so that domestic life becomes an ecosystem whose resources, risks, and moral rules are narrated through women's bodies, roles, and social positioning (3–6). The central claim, in other words, is that women in these proverbs

are not simply “represented”; they are placed inside a gendered moral ecology where matter food, adornment, containers, pests, and threats serves as the persuasive medium through which social order is made to feel natural, inevitable, and repeatable.

The primary corpus for this study consists of sixty women-focused Saudi proverbs drawn exclusively from the Saudi National Encyclopedia’s regional proverb archives (7), which curate local sayings as heritage discourse and thus provide a stable documentary base for analysis. Because the encyclopedia preserves proverbs alongside glosses that implicitly frame “what the proverb is for,” it also reveals which gendered judgments are considered representative enough to be archived as regional cultural memory, and which material images are treated as credible vehicles for moral instruction. The eco-folklore dimension of the corpus becomes visible the moment one attends to how often women are anchored to household infrastructures and domestic processes rather than to abstract virtues. A succinct example is “لا حرمة ولا برمة” (lā ḥurmah wa-lā burmah; “No wife and no cooking pot”), which condenses the household into a paired ecology of person and container, implying that domestic responsibility is inconceivable without women and without the material apparatus of cooking that women are presumed to manage. Another proverb anchors maternal value in the thermodynamics of care: “اللي أمه في الدار قرصه حار” (illī ummah fī ad-dār qurṣuh ḥār; “The one whose mother is in the house, his bread is hot”), where “hot bread” becomes the evidence of protection, intimacy, and reliable provisioning; after the proverb is stated, the English translation “The one whose mother is in the house, his bread is hot” functions as a theory of household security grounded in material warmth rather than in sentimental rhetoric. These are not incidental images. They enact what material ecocriticism calls the storied agency of matter: bread, pots, heat, and domestic routines do not merely decorate meaning but carry it, allowing social evaluation to borrow the credibility of material experience (3,4,8). For women-focused proverbs, borrowing is especially consequential because the domestic sphere is where women’s labor is both most necessary and most taken for granted; the proverb’s ecological imagery therefore becomes a way of naturalizing the extraction of care as if it were simply “how households work,” not a gendered distribution of obligations(5,6).

At the same time, women-focused Saudi proverbs do not operate only through praise or only through restriction; they frequently encode ambivalence, and that ambivalence is often expressed through nonhuman threats and contamination metaphors that transform social arrangements into

ecological hazards. Consider the proverb that captures the domestic violence of polygyny as an environmental danger rather than a theological institution: "عقربة في الغار ولا ضرة في الدار" (ʿaqrabah fī al-ghār wa-lā ḍarrah fī ad-dār; "A scorpion in the cave is better than a co-wife in the house"), which frames the co-wife as a more intolerable threat than a venomous creature; once the proverb is introduced, "A scorpion in the cave is better than a co-wife in the house" reads like a vernacular risk assessment in which the home becomes a site of chronic harm when patriarchal marriage produces rivalry as a structural condition. A related moral ecology appears in proverbs that treat "goodness" as materially compromised rather than morally pure, as in "حسنة وفيها ذباب" (ḥasnah wa-fīhā dhubāb; "A good thing with flies in it"), which recognizes value as mixed with contamination and trains speakers to judge whether the "flies" are tolerable; after stating it, "A good thing with flies in it" becomes a compressed ethic of ambivalence that is easily redirected toward women's reputations praise paired with suspicion because flies are culturally legible as both ordinary and repulsive. Even proverbs about "help" can become commentaries on intervention, gendered expertise, and unintended harm, as in "جاء يكحلها عماها" (jāʾ ykaḥḥilhā ʿamāhā; "He came to apply kohl and blinded her"), which makes bodily adornment the narrative machinery of failure; once present, "He came to apply kohl and blinded her" is a vernacular warning about meddling and repair that recruits a feminine-coded substance (kohl) to render the lesson memorable. These examples anticipate the article's broader argument: the proverb's nonhuman and material elements, scorpions, flies, and kohl are not neutral metaphors but ideological instruments that move gender norms into the domain of "natural" cause-and-effect, making social arrangements feel as self-validating as sting, spoilage, or blindness.

Building on scholarship that reads proverbs as pragmatic and ideological acts(9,10) and feminist discourse scholarship that tracks how gendered power is naturalized in everyday language (11,12), the article proposes "gendered moral ecology" as an analytic lens for Saudi women's paremiology: a way of naming how moral evaluation, household resource ethics, and gender hierarchy co-produce each other through matter. In dialogue with ecofeminist work that links domestic labor and environmental governance to broader logics of domination (5,6) and with emerging Saudi-focused proverb scholarship that demonstrates how proverb discourse encodes ideology in regional registers (13,14), the study asks three guiding questions: how do women-focused proverbs mobilize material objects and nonhuman life to stabilize judgments about women; what kinds of domestic environments and resource ethics do these proverbs imagine as "normal"; and where do

the proverbs preserve traces of critique especially around polygyny, wedding labor, and reputational surveillance through irony, hyperbole, and the language of exhaustion? Answering these questions matters because the proverb is not merely a record of past attitudes; it is an active cultural instrument that continues to shape what can be said, believed, and enforced in everyday life, particularly when women's bodies and choices are evaluated through the seemingly objective authority of ecological metaphors. The sections that follow therefore, situate the study within proverb studies, feminist discourse analysis, and environmental humanities scholarship, then detail the corpus and interpretive procedure, and finally present an analysis in which all sixty proverbs are integrated as primary evidence, each introduced once in Arabic with transliteration and English translation and then discussed thereafter through the English translation alone.

2. Research Objectives

2.1. General Objective

The general objective of this study is to conduct an ecocritical analysis of women-focused Saudi proverbs as a form of eco-folklore, exploring how they embed gender ideology within domestic material and environmental imaginaries to naturalize norms around women's roles, labor, and social positioning in Gulf cultural contexts.

2.2. Specific Objectives

The specific objectives of this study are as follows:

1. To examine how these proverbs utilize material objects and nonhuman elements (e.g., bread, cooking pots, kohl, henna, flies, and scorpions) to construct and reinforce gendered judgments regarding women's roles, labor, mobility, and moral accountability.
2. To investigate the portrayed domestic environments and resource ethics in the proverbs, pinpointing patterns of provisioning, stability, scarcity, contamination, and risk that conceptualize the household as a gendered moral ecology.
3. To identify and analyze elements of critique or ambivalence in the corpus, focusing on proverbs related to polygyny, wedding labor, reputational surveillance, and exhaustion, and assessing how irony, hyperbole, or material metaphors enable vernacular resistance while upholding normative discipline.

4. To synthesize proverb studies, feminist critical discourse analysis, and material ecocriticism to interpret Saudi proverbial discourse as eco-folklore, thereby enhancing insights into vernacular environmental knowledge, gender ideology, and domestic governance in the Gulf region.

These objectives direct the examination of a defined corpus of 60 women-focused Saudi proverbs sourced from the Saudi National Encyclopedia's regional archives (7), intending to reposition paremiology as an everyday discourse of ecological reasoning linked to gender norms.

3. Literature Review

Proverb studies have long treated the proverb as a distinctive discourse genre whose force lies less in its propositional content than in its social life: a proverb works because it is publicly recognizable, rhetorically "ready-made," and interactionally efficient, allowing speakers to compress experience into a transportable judgment(9,10). Cognitive and pragmatic approaches similarly emphasize that proverb meaning is not simply "in" the sentence; it is activated through situation, speaker intent, and shared cultural frames, which is why the same proverb can function as consolation in one context and coercion in another(10,15). This matters for women-focused proverb corpora because gender is often regulated not through explicit commands but through repeatable evaluations that appear neutral precisely because they are conventional. A proverb like "في القبال مراية وفي القفا مقص" (fī al-qibāl marāyah wa-fī al-qafā maqṣ; "A mirror in front and scissors behind") does not mention women lexically, yet in practice it can operate as a gendered technology of reputational surveillance when invoked in contexts where women bear disproportionate costs for gossip and social exposure; once introduced, "A mirror in front and scissors behind" becomes a compact model of social ecology where harm is intimate, hidden, and cutting, and where the listener is trained to treat everyday relations as environments that must be navigated with interpretive caution rather than trust. The relevance of proverb theory, then, is not merely definitional; it helps clarify why proverbs so often serve as instruments of gender governance: they circulate as a kind of cultural shortcut that replaces argument with recognizability and replaces evidence with inherited credibility (9). Yet proverb scholarship also warns against treating proverbs as static "beliefs," because their uptake is contingent and strategic; communities use proverbs to manage face, to legitimize decisions already made, and to settle moral disputes by

borrowing the authority of tradition (10). That contingency is especially important for the present study because the Saudi proverb corpus is not being approached as a transparent mirror of "Saudi attitudes toward women," but as a repertoire of discursive resources through which gendered norms are reproduced, negotiated, and sometimes ironically critiqued in the medium of ordinary speech.

A second body of scholarship, gender-focused proverb research across Arabic-speaking contexts, demonstrates that proverbial discourse often sustains patriarchal norms through recurring representational patterns: women are frequently linked to domesticity, sexuality, suspicion, and reputational fragility, while men are linked to authority, reason, and public value (16–18). Importantly, this scholarship shows that gender ideology in proverbs is not only a "negative portrayal" in the obvious sense; it also appears through what looks like praise, where women are valued primarily as mothers, caretakers, and stabilizers of the household, a pattern that aligns with the broader argument that "positive" depictions can still consolidate unequal role expectation. Comparative and regional studies on Arabic proverbs have used critical discourse analysis frameworks to demonstrate how evaluative adjectives, metaphors, and generalized claims normalize gender difference as if it were a natural order(17,19). Recent work has also examined how sexism can be perpetuated through proverb circulation as a cultural mechanism of "common sense," making the proverb a unit of ideological reproduction rather than merely a linguistic artefact (20). In the specific case of southern Arabian proverbs, Al-Asmari's study (21) is instructive because it frames gender representation as a discourse phenomenon shaped by ideology and cultural expectations, rather than as a mere list of stereotypes; this orientation supports the present project's insistence that women-focused proverbs should be read as discursive acts that organize social life. At the same time, scholarship on the translatability of women's proverbs and dialectal sayings shows that gendered meaning is frequently carried by culturally specific imagery objects, domestic practices, and kin terms that do not map neatly onto English without interpretive loss(22). This is directly relevant to the methodology of the present article, which retains Arabic and transliteration before translation precisely because proverb force often resides in vernacular texture, while the subsequent analytic discussion uses the English translation only to sustain coherence and to avoid repeating Arabic forms unnecessarily. The upshot of this gender-and-proverb scholarship is a clear baseline: women's proverbial representation is widely recognized as a site where ideology is condensed and normalized; what is still needed, however, is a framework

that explains why the condensation so often travels through matter objects, animals, substances and how that materiality functions as more than ornament in the production of gendered common sense.

That need is where feminist critical discourse analysis (FCDA) becomes methodologically decisive, because FCDA is designed to track how gendered power is naturalized through routine language forms that appear ordinary, humorous, or "just traditional"(11,12). FCDA attends to what proverbs do structurally: they generalize; they moralize; they stabilize binaries; they allocate blame; they frame some behaviors as shameful and others as respectable; and they accomplish these effects by presenting judgments as widely shared and therefore difficult to contest. In practice, proverbs often produce what looks like self-evidence through everyday metaphors that convert social relations into seemingly objective patterns, and this is one reason they are especially effective in regulating women's lives: they can discipline without overt coercion by rendering the judgment "already known." A proverb such as "بعد الكبرة جبة حمرة" (baʿd al-kibrah jubbah ḥamrāʾ; "After old age, a red robe") exemplifies how bodily display is policed through ridicule that feels culturally self-validating; once introduced, "After old age, a red robe" functions as a portable critique of women's self-fashioning, implying that certain forms of visibility become illegitimate with age and that the public eye has the right to mock a woman who violates the aesthetic boundaries assigned to her. The proverb's power lies in its compression: it does not need to explain why a "red robe" is inappropriate, because the cultural frame is presumed; it simply triggers the frame. Similarly, proverbs that encode suspicion and hidden harm without naming women explicitly become gendered tools in practice when deployed in contexts where women's reputations are treated as fragile resources; in these cases, FCDA helps prevent the analyst from mistaking "general" wording for neutral social function (11). Because the present article is interested not only in what is said about women but how women are governed through the everyday circulation of evaluative speech, FCDA provides a rigorous way to connect proverb form (generalization and portability) to social effect (discipline, normalization, ridicule, and sometimes counter-speech).

The environmental humanities and ecocriticism literature offer the conceptual bridge that turns "women's proverbs" into "eco-folklore," particularly through material ecocriticism and ecofeminist thought. Material ecocriticism argues that matter is not a passive background against

which meaning unfolds, but a participant in meaning-making; texts, genres, and discourses are saturated with "storied matter," where bodies, objects, and environments carry narratives of agency, risk, and value (3,4,8). Ecofeminist scholarship further insists that the governance of women's bodies and the governance of "nature" have historically been linked through shared logics of control, extraction, and hierarchical valuation, especially in domestic contexts where care labor, cleanliness, and provisioning become morally coded expectations rather than recognized work (5,6). These frameworks are highly productive for proverb analysis because proverbs frequently recruit material and nonhuman imagery precisely when they want to make a social claim feel inevitable. Consider "كل همّة ولها قمّة" (kull hammah wa-lahā qammah; "Every creature has its match"), a saying that borrows the ecological logic of pairing and distribution nothing is without an ecological niche and applies it to human social fate; once introduced, "Every creature has its match" becomes a moral ecology of inevitability that can be used as consolation or as insult depending on context, and in gendered settings it can quietly naturalize the idea that women's marital outcomes are governed by fate-like "matching" rather than by structural inequalities. Or consider a proverb whose imagery is domestic and infrastructural rather than "nature scenic": "العرس لاثنين والتعب لألفين" (al-ʿurs li-ithnayn wa-t-taʿab li-alfayn; "The wedding is for two, and the exhaustion is for two thousand"), which compresses a whole political economy of ritual into a numerical contrast; once stated, "The wedding is for two, and the exhaustion is for two thousand" becomes an ecofeminist insight in vernacular form because it exposes how communal celebration depends on distributed labor often feminized labor while benefit is concentrated in the couple's social prestige. Material ecocriticism clarifies why such a proverb persuades: "exhaustion" is not abstract; it is bodily depletion, time, heat, and effort, and those material realities become the grounds on which social critique is made legible (3,4,8). What these frameworks add, therefore, is not a vague insistence that "proverbs mention nature," but a precise claim that material and nonhuman elements function as the credibility engine of gendered judgment: they make norms feel like ecology like heat, fatigue, matching, scarcity, contamination rather than like ideology.

Saudi-focused proverb scholarship provides the immediate disciplinary context for the present study, particularly research that treats Saudi proverbs as culturally specific discourse with regional variation, ideological content, and translation challenges. Work on Saudi Hijazi proverbs has explicitly connected proverb discourse to power and gender, showing that regional proverb repertoires can carry ideologically structured representations rather than merely "folk color" (13).

Comparative work on Egyptian and Saudi proverbs has also demonstrated that proverb semantics can be mapped systematically through linguistic relations such as antonymy, indicating that Saudi proverbial language can be analyzed with formal rigor rather than only with impressionistic cultural commentary (14). Translation studies on Najdi proverbs similarly emphasizes that dialectal proverbs embed cultural cues that require interpretive strategies to preserve meaning in English, reinforcing the methodological decision to retain Arabic and transliteration alongside translation in the present article (23,24). Eco-linguistic approaches to Saudi regional proverbs, including research on animal representation in Al-Bahah proverb discourse, further demonstrate that Saudi proverbs can be read as environmental texts at the level of metaphor and value orientation, not merely as "sayings about society" (25). Yet despite these advances, two gaps remain visible when the literature is considered as a whole. The first is a corpus gap: many studies focus on a single region, a single semantic field (animals, body parts, advice), or a narrow selection of examples, which can limit claims about how women-proverbs operate as a broader discursive ecology. The second is a conceptual gap: even when gender ideology is analyzed carefully, the material and environmental imagery through which that ideology is made persuasive often remains under-theorized. This article addresses both gaps by analyzing a bounded corpus of sixty women-focused Saudi proverbs curated in the Saudi National Encyclopedia's regional proverb archives (7), and by reading them through an integrated framework that holds together proverb theory (portability and pragmatics), feminist discourse analysis (naturalization of gender power), and material ecocriticism/eco-folklore (matter and nonhuman imagery as carriers of moral force). The transition from scholarship to method is therefore straightforward: because proverbs do ideological work by traveling through repeatable forms, and because women-focused proverbs frequently travel through matter, the next section details how the sixty-proverb corpus is defined, how "women-focused" is operationalized without flattening context, and how the analysis is structured to integrate each proverb once in Arabic with transliteration and English translation before interpreting it thereafter through the English translation alone.

4. Theoretical and Analytical Frameworks

This study employs an interdisciplinary methodology to interpret women-focused Saudi proverbs as eco-folklore, a vernacular tradition in which gender ideology is embedded within domestic material and ecological imaginaries. By weaving together insights from proverb studies, feminist

critical discourse analysis, and environmental humanities, the analysis situates proverbs not only as linguistic artifacts but also as cultural technologies that mediate the relationship between gender, morality, and material life.

i. **Proverbs as Discursive Acts:** Proverbs are approached here through the lens of pragmatic and cognitive proverb studies, which emphasize their dynamic and context-dependent nature. Rather than treating them as static repositories of belief, this framework highlights how proverbs function as portable verdicts, short, recognizable utterances that can be mobilized in everyday situations to justify, warn, or critique. Their persuasive power lies in their portability, recognizability, and adaptability, enabling them to circulate across generations and social settings while retaining authority (3,4,10). In the Saudi context, women-focused proverbs often condense communal judgments into a single phrase, making them difficult to contest in ordinary conversation. This portability transforms them into disciplinary technologies, shaping expectations about women’s labor, morality, and reputation through repetition and familiarity.
ii. **Feminist Critical Discourse Analysis (FCDA):** This lens examines how gendered power relations are normalized through everyday language that often seems benign, traditional, or witty. The proverbs are scrutinized for their discursive mechanisms such as generalization, moral framing, binary oppositions, and allocation of responsibility, which condense ideology into apparent "common sense" and disproportionately govern women's labor, reputation, and autonomy (3;4).
iii. **Material Ecocriticism and Ecofeminism:** These environmental humanities perspectives enable the reading of proverbs as eco-folklore. Material ecocriticism highlights the "storied agency" of matter, positioning objects and processes as active contributors to meaning rather than mere illustrations (3,11,20). Ecofeminism connects parallel logics of domination over women and the environment, especially in household settings involving extraction, caregiving, and hierarchical valuation (4,6).

These frameworks are enriched by insights from Saudi and broader Arabic proverb research, which underscore ideological content, cultural specificity, and recurring gendered representations (e.g., 11; 18). Through this synthesis, the study reconceptualizes Saudi women's paremiology as a

"gendered moral ecology": a vernacular discourse in which moral evaluation, domestic resource ethics, and gender hierarchy intertwine via material objects and nonhuman elements.

5. Methodology

5.1.Corpus Selection and Assembly

This study analyzes a bounded corpus of sixty women-focused Saudi proverbs drawn exclusively from the regional proverb archives of the *Encyclopedia of the Kingdom of Saudi Arabia* (Mawsūʿat al-Mamlakah al-ʿArabīyah al-Saʿūdīyah), a project of the King Abdulaziz Public Library (2007). All proverbs analyzed are treated as "Saudi" in the archival sense of national documentation, while recognizing that some items may also circulate transregionally across Arabic-speaking communities in variant forms. The decision to work with this encyclopedia-based corpus is methodological rather than merely convenient: the encyclopedia curates proverbs as heritage discourse, providing a stable archive of sayings that are presented as regionally recognizable and culturally representative, which allows the analysis to focus on how gendered judgments become "collectable" and institutionally legible as vernacular knowledge. The corpus was assembled by extracting proverbs that either (a) explicitly reference women through kinship and gender terms (mother, wife, co-wife, bride, mother-in-law, and other direct feminine markers), or (b) are routinely mobilized in the encyclopedia's own framing as commentary on women's reputation, domestic competence, marital relations, or gendered visibility, even when the proverb's wording appears more general. This inclusion rule is necessary because women's governance in proverbial discourse often occurs through apparently universal statements that function asymmetrically in practice; by treating "women-focused" as a functional category rather than a purely lexical one, the corpus remains sensitive to how gender is produced as an interpretive target. At the same time, the corpus is deliberately restricted to sixty items to maintain analytic depth: the goal is not to provide a catalog but to offer a close, theory-informed reading in which each proverb can be contextualized, interpreted, and connected to the article's argument about gendered moral ecology without collapsing into repetitive paraphrase.

5.2.Analytic Design

The study combines feminist critical discourse analysis with material ecocriticism and ecofeminist conceptual vocabulary to examine how proverbial language stabilizes gendered norms by recruiting matter and nonhuman imagery as moral evidence (4,5,26). It employs a sequential qualitative split design (mixed-methods within a qualitative approach), enhancing rigor by balancing breadth (pattern identification) and depth (interpretive analysis). In the first phase, each proverb was systematically coded along three dimensions: (1) primary social domain (e.g., kinship/maternal authority, marriage/marital conflict, domestic labor, reputation/surveillance, bodily aesthetics); (2) dominant discourse function (e.g., warning, ridicule, instruction, justification, consolation, exposure of hypocrisy), with attention to generalized grammar that produces inevitability; and (3) material or ecological anchor (e.g., food/heat, containers, pests/animals, textiles, bodily substances like kohl/henna, spatial environments). These codes were cross-tabulated to identify recurring patterns, such as how responsibility is narrated through infrastructure or risk through contamination. In the second phase, the coded patterns informed iterative close readings, where the proverbs themselves serve as primary evidence; scholarship clarifies conceptual tools rather than replacing close reading.

5.3.Translation Approach

Translating dialectal Saudi proverbs into English poses significant challenges due to cultural specificity, metaphorical density, and the loss of vernacular texture (e.g., material references to burmah pots or kohl that carry ideological weight). Literal translations often render sayings opaque, odd, or culturally distorted for English readers, failing to convey the ecological and gendered imaginaries (e.g., warmth as care or contamination as moral risk). Excessive domestication seeking direct English proverb equivalents risks erasing source-culture elements, while over-reliance on cultural substitution can dilute the proverb's persuasive force in its original domestic context.

This study therefore, adopts a functional ("working") translation strategy that prioritizes discursive force, material imagery, and ecological logic over word-for-word equivalence, aligning with principles of dynamic equivalence (27) and communicative translation (26) Dynamic/communicative approaches emphasize naturalness and equivalent effect in the target language, ensuring the proverb evokes a similar response in English readers while preserving

cultural communicativeness particularly the "storied matter" (e.g., preserving "hot bread" for sensory warmth and provisioning rather than abstracting to "care"). This avoids the pitfalls of formal/literal equivalence, which prioritizes source-language form but often distorts cultural nuances in Arabic proverbs (e.g., religious or regional connotations lost in rigid rendering).

Each proverb appears once in the article as: Arabic script (transliteration; English working translation), with glosses where needed for clarity (e.g., explaining nonhuman threats like scorpions or flies). Subsequent discussion refers only to the English translation for coherence and readability. Transliteration follows scholarly conventions (ʿ for ع, ḥ for ح, etc.), acknowledging regional variation. This hybrid approach, combining communicative naturalness with selective literal retention of key material anchors, ensures the proverb's ideological medium (heat, depletion, contamination) remains visible and analyzable, facilitating cross-cultural communication without full domestication.

5.4.Presentation Protocol and Limitations

Each proverb appears once in the article in the following order: Arabic proverb (transliteration; English translation). Immediately after that first appearance, the discussion refers to the proverb using only the English translation to maintain readability and avoid repetition.

The study's limitations are methodologically acknowledged: The corpus is archival, not ethnographic, so findings address discursive potentials rather than usage frequency. Curatorial biases may favor certain perspectives. Translations risk nuance loss, though material anchors are preserved. The qualitative approach lacks quantification; future work could use computational methods.

6. Results/Analysis

The following analysis presents the findings from the close reading of the sixty women-focused Saudi proverbs drawn from the Saudi National Encyclopedia's regional archives (7). Organized thematically, the discussion reveals how these sayings construct a gendered moral ecology in which material objects, nonhuman elements, and domestic processes serve as the primary vehicles for evaluating women's roles, labor, mobility, and moral worth. Each subsection examines a key domain of domestic life maternal authority and kinship, marriage and desire, domestic work and

provisioning, visibility and adornment, and additional relational patterns while integrating all relevant proverbs as primary evidence. By retaining the Arabic original, transliteration, and English working translation at first mention and thereafter referring only to the English translation, the analysis preserves both cultural specificity and readability.

6.1.Maternal Authority, Kinship, and the Household as an Ecological Infrastructure

In this subsection, the proverbs centered on maternal figures and kinship ties are examined as foundational elements of the domestic moral ecology, where women particularly mothers are positioned as indispensable anchors of household stability, protection, and moral continuity. These sayings frequently mobilize sensory and material metaphors, such as warmth, presence, and bodily processes, to frame maternal roles not merely as social obligations but as ecological imperatives that sustain the family's "infrastructure" against depletion, dishonor, or instability. This thematic cluster underscores the corpus's broader ambivalence: while ostensibly valorizing mothers as sources of irreplaceable care and authority, the proverbs often reinforce gendered hierarchies by tying women's value to exhaustive provisioning and filial obedience, thereby naturalizing unequal distributions of emotional and material labor within the home.

Across the corpus, maternal presence emerges as a foundational domestic ecology, embodying an infrastructure of heat, protection, honor, and moral legitimacy that renders care both natural and indispensable. Proverbs such as "اللي أمه في الدار قرصه حار" (alli ummuh fī ad-dār qirṣah ḥār; "If his mother is at home, his bite (of bread) is hot he's well cared for.") ground care in tangible sensory proofs like the warmth of fresh bread, prioritizing material evidence over abstract sentiment. This indispensability extends to sayings like "اللي ماله أم حاله يغم" (illī mā lah umm ḥālah yighamm; "Whoever has no mother, his condition grieves (he lacks support)."), "اللي ما له أم، ما له عِزّ" (illī mā lah um, mā lah ʿizz; "Whoever has no mother has no honor/support."), and "متفدية غير أمك كذابة" (mutfaddiya ghayr ummak kadhābah; "Any nurse/comforter other than your mother is pretending."), which positions mothering as an irreplaceable source of social status and reliable support, rendering alternative care ecologically unstable. Maternal affection further reshapes perception, as seen in "القرد في عين أمّه غزال" (al-qird fī ʿayn ummuh ghazāl; "In his mother's eyes, a monkey is a gazelle.") and "الأم تحب ولدها ولو كان قردًا" (al-umm tuḥibb waladahā walaw kāna qirdan; "A mother loves her child even if he's a monkey."), where unconditional love creates

interpretive distortions that hold moral authority despite conflicting with external judgments. Authority reaches sacral heights in "الأمّ إذا رضيت رضى الله" (al-umm idhā raḍiyat raḍā Allāh; "When a mother is pleased, God is pleased."), routing divine favor through the domestic realm and elevating filial obedience to an environmental imperative. Maternal lines serve as epistemic anchors in "إذا رأيت أمي، رأيت عبري" (idhā raʾayt ummī, raʾayt ʿabrī; "When I see my mother, I see my lesson/example.") and "مع أمهاتها ولو ضلعت" (maʿ ummahātihā walaw ḍalaʿat; "With its mothers—even if it limps (stick with one's maternal line)."), portraying them as the most stable habitat even amid imperfection. Gendered roles are partitioned in "أنا أمكم حميتكم، وأنا أبوكم أكلتكم" (anā ummakum ḥamaytkum, wa-anā abūkum akaltkum; "I'm your mother: I protected you; I'm your father: I fed you."), Framing protection and sustenance as complementary ecosystems. Shared human origins appear in "كلنا عوال تسعة أشهر" (kullunā ʿawāl tisʿat ashhur; "We're all (born) after nine months (human equality)."), using gestation as a leveling force, while "ابن أصول ما يعيّب أمه" (ibn uṣūl mā yʿayyib ummah; "A well-bred man doesn't shame his mother.") treats maternal honor as a depletable public resource. Collectively, these proverbs construct the household as a moral ecosystem stabilized through women's maternal roles, transforming mothers into essential environmental foundations.

6.2. Marriage, Desire, and the Distribution of Risk in Gendered Domestic Worlds

Turning from the stabilizing imagery of motherhood to the more volatile domain of marriage, the proverbs reveal the household as a site of negotiated risk, desire, and structural inequality. Partner selection, sexual propriety, polygyny, and wedding rituals are framed through ecological metaphors of hazard, scarcity, and lineage inheritance. Women's value and vulnerability are assessed primarily through their reproductive potential and reputational capital, while marital arrangements especially polygyny and communal wedding labor are frequently critiqued through comparisons to natural dangers and disproportionate toil. These sayings thereby expose marriage not as a private bond but as a governed distribution of domestic risk in which women bear the greatest moral and material burdens.

While maternal proverbs depict the household as stable, those on marriage reveal its volatility, negotiating women's value and vulnerability via kinship selection, polygyny, propriety, and unequal labor. Partner choice is framed as lineage risk assessment in the triad " اسأل عن أمها قبل ما

تضمها" (isʾal ʿan umm-hā qabl mā tḍumm-hā; "Ask about her mother before you marry/embrace her."), its variants, emphasizing maternal reputation as inherited ecology over individual agency. Reproductive outcomes tie to behavior in "من استحى من بنت عمه ما جابت ولد" (man istaḥā min bint ʿammih mā jābat walad; "He who is too shy with his cousin won't have children."), while desire borrows animal instincts in "عنز البلاد ما تبغى إلا التيس الغريب" (ʿanz al-bilād mā tabghā illā at-tays al-gharīb; "The local doe wants only the foreign buck (preferring the outsider)."), naturalizing preference for outsiders. Propriety demands extreme sacrifice in "تجوع الحرة ولا تأكل بثدييها" (tijūʿ al-ḥurra walā taʾkul bi-thadyayhā; "A free woman would rather starve than eat by selling her body."), equating chastity with survival priority. Marital privacy shields dynamics in " ما يدري ما بين الحليلين إلا الله" (mā yidrī mā bayn al-ḥalīlayn illā Allāh; "No one knows what's between spouses except God."), yet polygyny draws sharp critique in "عقربة في الغار ولا ضرة في الدار" (ʿaqrabah fī al-ghār wa-lā ḍarrah fī ad-dār; "A scorpion in the cave is better than a co-wife in the house."), portraying co-wives as greater domestic hazards than natural threats. Weddings expose labor inequities in " العرس لاثنين والتعب لألفين" (al-ʿurs li-ithnayn wa-t-taʿab li-alfayn; "The wedding is for two; the toil is for two thousand."), highlighting feminized toil distributed widely for concentrated benefit. Timing failures appear in "يوم راحت الدفوف جت الخاملة تشوف" (yawm rāḥat ad-dufūf jāt al-khāmilah tshūf; "When the drums are gone, the lazy one comes to watch (too late)."). Overall, marriage functions as governance, assessing risks through maternal lines, constraining dignity, framing polygyny as hazard, and revealing rituals as exploitative labor ecologies.

6.3. Domestic Work, Provisioning, and Storied Matter as the Medium of Gender Evaluation

A large cluster of proverbs evaluates women's worth almost exclusively through their mastery of everyday domestic objects and processes. Bread, cooking pots, dyes, and other household implements become "storied matter" that carries moral judgment: competence in their use signals virtue and responsibility, while failure or absence signals chaos and inadequacy. Through repetition and variation, these sayings naturalize women's identification with household infrastructure, presenting provisioning and maintenance labor as an ecological imperative rather

than a negotiable division of work. The material objects themselves thus function as the primary medium through which women are praised, blamed, or held accountable.

Domestic competence is repeatedly moralized through everyday objects, with variants reinforcing norms via repetition. The Riflah cluster—"خبزك يا الرفلة وكليه" (khubzik yā al-riflah w-kulīh; "Eat the bread you baked, Riflah.") and variants, plus "خبزك خبز أمك" (khubzik khubz ummak; "Your bread is your mother's bread.")—enforces accountability and maternal ties in provisioning. Women merge with infrastructure in "لا حرمة ولا برمة" (lā ḥurmah wa-lā burmah; "No wife and no cooking pot (no responsibilities)."), naturalizing their role in household survival. Dysfunction chains emerge in "عمياء تخضب مجنونة وصنجا تتسمع لهم" (ʿamyāʾ takhḍib majnūnah wa-ṣanjāʾ tatasammaʿ lahum; "A blind woman dyes a mad woman, and a deaf one listens to them (incompetence all around)."), dramatizing failure through mismatched tasks. Hierarchy blocks reversal in "العنز لا تعلّم أمها الرضاعة" (al-ʿanz lā tuʿallim ummahā ar-raḍāʿah; "A young goat doesn't teach its mother how to nurse."). Collective need is acknowledged yet feminized in " يد واحدة ما تصفق، وامرأة واحدة ما تدبّر بيت" (yad wāḥidah mā tuṣaffiq, wa-imraʾah wāḥidah mā tudabbir bayt; "One hand doesn't clap; one woman alone can't run a household (need help)."). Scarcity materializes through women's objects in "ما عنده إلا ما عند جدتي" (mā ʿindah illā mā ʿind jaddatī; "He has nothing but what my grandmother has.") and "إيش معش يا مستورة؟ قالت: حُقّ وقارورة" (īsh maʿsh yā mastūrah? qālat: ḥuq wa-qārūrah; "'What do you have, Mastūrah?' 'A tin and a bottle' (utterly broke)."). Resource constraints highlight in "قال سبّري يا جارية قالت قرّب يا سيدي" (qāl sabbirī yā jāriyah qālat qarrib yā sayyidī; "He said 'Prepare…'; she said 'Bring (the supplies), sir' (means require means)."). Thus, storied matter bread, pots, and dyes serve as the primary medium for praising, blaming, or obligating women's domestic labor.

6.4. Visibility, Adornment, Reputation, and Moral Regulation Through Sensory Ecologies

Women's bodies and self-presentation emerge as another critical site of moral regulation, where adornments (kohl, henna, bangles) and sensory experiences (staining, visibility, rumor) serve as metaphors for reputational fragility and social surveillance. These proverbs shift evaluation from

internal character to external appearance and spatial positioning, often using material transformations (reddening, depletion, deception) to warn against excess, meddling, or nonconformity. The domestic sphere is reconceived as a sensory ecology in which women's visibility is both a resource and a risk, subject to constant interpretation and discipline by the communal gaze.

Women's evaluation shifts to sensory realms, where adornments like kohl and henna bear moral loads, and spatial binaries foster suspicion. Risky intervention appears in "جاء يكحلها عماها" (jāʾ ykaḥḥilhā ʿamāhā; "He came to apply kohl and blinded her."), using feminine aesthetics to warn of harm. Essence trumps effort in "الزين زين لو قعد من النوم، والشين شين لو كحل عيونه" (az-zayn zayn law qaʿad min an-nawm, wa-sh-shīn shīn law kaḥḥal ʿuyūnah; "Beauty is beauty even out of bed; ugliness is ugliness even with kohl."), policing beautification while essentializing worth. Vulnerability intimates in "يسرق الكحل من العين" (yasriq al-kuḥl min al-ʿayn; "He'd steal kohl from the eye (extremely crafty)."). Rumor rapidity fuses with staining in "ما سارع ذا الحنا لمع" (mā sāriʿ dhā al-ḥinnā lamaʿ; "How fast henna reddens (how fast news spreads)."). Compliance demands in "اللي عاجبه الكحل يتكحل واللي موب عاجبه يرحل" (illī ʿājbah al-kuḥl ytakḥal wa-illī mūb ʿājbah yirḥal; "If you like the kohl, apply it; if not, leave."). Depletion teaches in "جبال الكحل تخليها المراود" (jibāl al-kuḥl takhallīhā al-marāwid; "Mountains of kohl are worn down by the applicator (use consumes resources)."). Deception warns in "خلاخل والبلا من داخل" (khalākhil wa-l-balā min dākhil; "Bangles on the outside, trouble within (appearances deceive)."). Relapse mnemonics use named figures in "عادت حليمة لعادتها القديمة" (ʿādat Ḥalīmah li-ʿādatihā al-qadīmah; "Halīmah returned to her old habit.") and "تيتي تيتي زي ما رحتي جيتي" (tītī tītī zayy mā raḥtī jītī; "Tītī, Tītī—just as you went, you came back (no change)."). Inescapability states in "ما فيه يا أمي ارحميني" (mā fīh yā ummī irḥamīnī; "There's no 'Mother, have mercy on me' (no escape from consequences)."). Confinement mandates in "مكان المرأة بيتها" (makān al-marʾah baytuhā; "A woman's place is her home.") and dependence in "المرأة من رجالها، والخيل من ركابها" (al-marʾah min rijālihā, wa-l-khayl min rukābihā; "A woman is known by her men; horses by their riders."). Father-daughter complexities arise in "كل فتاة بأبيها معجبة" (kull fatāh bi-abīhā muʿjabah; "Every girl is enamored of her father."), " البنت سرّ أبوها" (al-bint sirr abūhā; "A daughter is her father's secret/treasure."), and "من دارى ببنته ما نكحت" (man dārā bi-bintahā mā nakaḥat; "Whoever over-shelters his daughter will not marry her off."). Stability burdens women in "إذا صلحت المرأة صلح البيت" (idhā ṣalaḥat al-marʾah ṣalaḥ al-bayt; "If the

woman is upright, the home is upright.") and related sayings praising wise or righteous wives as treasures or protectors. This ambivalence positions women as vital yet regulated through material and sensory discipline.

6.5. Additional Patterns in Domestic Moral Ecology

The remaining proverbs deepen and nuance the gendered moral ecology by addressing relational asymmetries, transitional dependencies, bodily imperatives, and affective instability. Stepmotherhood, pregnancy cravings, wavering commitment, and self-inflicted trouble are rendered through material and kinship metaphors that highlight uneven investment and inescapable consequence. Although less numerous, these sayings reinforce women's structural centrality to household legitimacy while illustrating how relational rhythms care, desire, exhaustion, and reversal are narrated through women's bodies and roles.

Remaining proverbs deepen the domestic moral ecology by addressing uneven care, self-inflicted harm, transitional dependencies, bodily demands, and affective shifts. Stepmotherly effort dilutes in "دهن مرة أبو" (dahn marrat abū; "(It's) the work of a stepmother (half-hearted workmanship)."), mapping kinship to variable investment. Self-caused trouble contaminates in " يا من شراله من حلاله علة" (yā man sharālah min ḥalālah ʿillah; "O one who bought himself trouble with his own money."). Adulthood policing critiques immaturity in "بغت ترزق وعيا أبوها" (baghat tarzq wa-ʿiyā abūhā; "She wanted children but (still) behaves like her father's dependent."). Maternal capital costs in "وش أنا مخلي أمي تروس له" (wush anā mukhallī ummī trūs lah; "Why would I let my mother go beg/mediate for him?"). Pregnancy cravings compel in "شهوة نسا" (shahwat nisā; "A woman's craving (especially pregnancy craving): an urge that won't be refused."). Wives as assets declare in "راس ماله أم عياله" (rās mālah umm ʿiyālah; "His real capital is the mother of his children (his wife)."). Oscillation wavers in "حيَت أمك ماتت أمك" (ḥayyat ummak mātat ummak; "Your mother lived—your mother died (an undecided, wavering state)."), and excess reverses in "أمّ الضحكة تبكيك" (umm aḍ-ḍaḥkah tabkīk; "The 'mother' of laughter will make you cry (excessive joy ends in sorrow)."). Together, these extend the ecology to uneven relational rhythms, reinforcing women's centrality in household legitimacy.

Taken together, the thematic patterns identified across the sixty proverbs demonstrate a remarkably cohesive vernacular system for reasoning about gender through domestic materiality and ecological imagery. Rather than offering isolated moral lessons, the corpus functions as an interconnected repertoire in which objects, substances, animals, and processes lend tangible credibility to judgments about women's labor, reputation, mobility, and worth. The consistent ambivalence praise intertwined with discipline, valorization shadowed by extraction emerges not as contradiction but as a structural feature of this gendered moral ecology. The Discussion that follows synthesizes these findings, situates them within existing scholarship, and explores their broader implications for ecocritical, feminist, and Gulf cultural studies.

7. Discussion

When examined as a unified corpus from the Results/Analysis section, the women-focused proverbs archived in the Saudi National Encyclopedia's regional collections (7) form a cohesive system of moral reasoning. In this system, material life does not simply illustrate gender ideology; it provides the tangible evidence that renders gendered judgments self-evident and easily transferable across everyday situations. Proverbs function as ready-to-use tools in social interactions, deriving their power from familiarity and repeatability rather than rigorous proof. They borrow authority from routine experiences such as heating bread, handling cooking vessels, bearing exhaustion, evading dangers, or interpreting appearances to present social norms as inevitable environmental realities.

Central to this corpus is the consistent use of domestic objects and nonhuman elements as the framework for evaluating women. Praise for women typically ties their worth to effective provisioning, household order, and resource management. In contrast, warnings, mockery, or restrictions often employ metaphors of pollution, closeness to harm, resource exhaustion, or concealed damage. This duality creates the repertoire's characteristic ambivalence: seemingly complementary proverbs heighten women's obligations by linking their value to flawless domestic oversight, while cautionary ones curb their freedom, visibility, and reputational control through sensory and spatial frameworks that portray household equilibrium as contingent on female conformity.

The broader significance lies in viewing women's paremiology as more than mere representation of gender; it serves as a vernacular repository of environmental logic applied to domestic morality. Here, household sustainability is conceived as an ethical economy, with women cast as its chief overseers, absorbers of strain, and absorbers of risk.

This material-ecological lens reveals why the proverbs resist binary categorization into those that "honor" or "disparage" women. Many who exalt motherhood or the virtuous wife simultaneously enforce the extraction of labor and care, naturalizing these as prerequisites for household viability and equating any shortfall with total domestic collapse. Conversely, proverbs posing as neutral life lessons often serve gendered purposes in application, since the burdens they highlight rumor, distrust, reputational damage, or public scrutiny, fall disproportionately on women, making "universal" advice operate unevenly.

The study's key contribution is illuminating these dynamics without idealizing proverbs as pure cultural heritage or reducing them to outright bias. Rather, it treats the collection as a flexible discursive framework that upholds hierarchies yet occasionally retains subtle critiques, particularly regarding arrangements that inflict ongoing hardship, fatigue, or instability. The ecological metaphors are not ornamental; they persuade by shifting contested gender norms into the realm of embodied causality, where social structures appear as straightforward as natural processes.

Situating these insights in relation to prior work, the analysis builds on proverb scholarship's emphasis on contextual and pragmatic meaning by demonstrating that, in women-centred sayings, such pragmatics are inextricably tied to materiality. The proverb's situational relevance often stems from aligning social issues with palpable material events. From a feminist discourse perspective, this alignment constitutes a potent naturalizing strategy, distilling intricate power relations into unquestionable insights conveyed via objects and routines that seldom provoke challenge.

The approach also advances Saudi-specific proverb research, which has recognized regional variations and ideological content, by urging deeper attention to domestic rather than scenic or wild materiality as the core archive for gender norms. It advocates broadening the notion of "environment" in Arabic proverbs to encompass the household and the body: sites of ongoing

resource negotiation, proximate dangers, and regulated visibility through adornment and spatial boundaries. This expansion is particularly valuable for ecocritical scholarship in Gulf studies, as it locates everyday environmental knowledge in concise oral forms, where it intertwines with gender ideology via the textures of home life.

8. Limitations

The study's limitations are noteworthy and help define its scope. First, the corpus derives from an encyclopedic archive rather than empirical records of current usage. Consequently, the findings address potential discourses, underlying logics, and rhetorical possibilities within the selected sayings, but not their frequency, demographic patterns, or real-time conversational impacts today.

Second, the encyclopedia's curation involves institutional choices, selection, glossing, and categorization of regional heritage that shape interpretation and may favor particular ethical perspectives. While the analysis incorporates this curation as part of its focus on how proverbs become "official" Saudi knowledge, it restricts access to fuller contemporary diversity, including how younger or female speakers might challenge, subvert, parody, or reject these sayings.

Third, the methodological choice to introduce Arabic and transliteration once before relying on English translations ensures readability but risks losing nuances in connotation, dialectal resonance, rhythmic effects, or context-specific implications. Although the translations prioritize retaining material-ecological meanings, subsequent research could include detailed commentary on challenging items or analyses that foreground dialect where essential.

Fourth, the qualitative, theory-guided interpretation relies on close reading and thematic grouping rather than quantitative measures. Future complementary work might employ computational methods to quantify recurring motifs (objects, animals, processes) in larger collections or comparative studies to assess whether the observed domestic-ecological focus is unique to women-focused proverbs versus those centered on men or neutral topics.

These constraints highlight promising directions for further inquiry: ethnographic recordings of actual proverb use to capture deployment dynamics; examinations of online repurposing in memes, threads, or videos where authority may be questioned or reinforced rapidly; and regional

comparisons to trace how shifts in imagery align with evolving household structures and women's roles. Ultimately, the findings and archival boundaries underscore a dual insight: proverbs actively supply mobile frameworks for evaluating and regulating domestic existence, often rendering gender inequities as innate as warmth, exhaustion, venom, or blemish. Acknowledging this process enables scholarship to view women's paremiology as a contextualized discourse of household ecological governance open to ongoing examination in contemporary practice.

9. Conclusion

This article has argued that women-focused Saudi proverbs, as curated in the Saudi National Encyclopedia's regional proverb archives (7), can be read as eco-folklore: a repertoire of condensed judgments in which gender norms are repeatedly made persuasive through material and environmental imaginaries rather than through abstract moral reasoning alone. By tracing how the corpus routes evaluation through household infrastructures and nonhuman figures bread warmth as proof of care, pots as shorthand for responsibility, flies and scorpions as calibrations of risk, and kohl/henna as technologies of visibility the study has shown that the proverb's authority is partly ecological: it borrows the credibility of matter and causality to make social expectations feel inevitable. Read through feminist critical discourse analysis, this ecological credibility helps explain the corpus's characteristic ambivalence: women are praised as the conditions of domestic stability and familial protection while simultaneously disciplined through reputational surveillance, spatial restriction, and essentialist judgments about appearance and worth (3; 4). From an ecofeminist and material ecocritical perspective, the same pattern reveals a moral economy in which women's care labor is positioned as natural infrastructure—expected, extracted, and rarely framed as labor while the domestic sphere becomes the primary ecosystem through which sustainability, scarcity, and social order are narrated (3,4,6).

The contribution of this approach is conceptual and methodological. Conceptually, it reframes Saudi women's paremiology as "gendered moral ecology," emphasizing that proverb discourse does not simply describe women but situates them within a system of resources, hazards, and obligations made legible through storied matter and nonhuman metaphors. Methodologically, it demonstrates how an encyclopedia-based proverb archive can be analyzed as a coherent corpus without reducing proverbs to static beliefs: by integrating each proverb as primary evidence and reading it as an interaction-ready device that can discipline, justify, ridicule, or critique depending

on context, the study treats proverb form as a cultural technology rather than a folkloric ornament (3,5). Taken together, these findings suggest that ecocritical inquiry in Gulf cultural studies can profitably move beyond long-form narratives to the smallest vernacular genres, where environmental reasoning appears not as landscape description but as domestic and bodily process. Future research can strengthen this intervention by pairing the encyclopedia archive with contemporary usage data spoken interaction, women's oral histories, and digital circulation to examine how these ecological metaphors are being maintained, reinterpreted, resisted, or repurposed under changing social conditions and gender regimes, while preserving the central insight that proverb authority often works by turning ideology into something that feels as obvious as heat, sting, spoilage, or depletion.

10. Recommendations and Implications

The findings of this study carry significant implications for multiple fields while opening avenues for practical and scholarly advancement. Conceptually, reframing Saudi women-focused proverbs as a form of eco-folklore vernacular expressions that embed gender ideology within domestic material and ecological processes broadens ecocritical and feminist approaches to Arabic oral traditions. By highlighting how everyday objects (bread, pots, kohl) and nonhuman elements (flies, scorpions) serve as persuasive tools for naturalizing gendered responsibilities, the analysis reveals that environmental reasoning in Gulf contexts often operates at the intimate scale of the household rather than the expansive landscape. This insight challenges dominant ecocritical tendencies to prioritize "wild" or rural nature, demonstrating instead that domestic sustainability, resource scarcity, and risk management are key sites where gender and ecology intersect. For feminist scholarship, the persistent ambivalence identified where praise for women as household "builders" or "treasures" simultaneously intensifies the extraction of care labor underscores how seemingly affirmative representations can reinforce patriarchal structures, aligning with broader ecofeminist critiques of domination logics that link control over women and nature.

In Gulf cultural studies, the study contributes to a more nuanced understanding of proverbial discourse as an active mechanism of moral governance rather than a passive heritage. Saudi proverbs, as archived and circulated, do not merely reflect attitudes toward women; they provide portable templates that continue to shape everyday evaluations of labor, mobility, reputation, and

marital arrangements. Recognizing their ecological framing helps explain their enduring persuasiveness: by borrowing the apparent inevitability of material processes (warmth fading, resources depleting, stains spreading), these sayings make contested norms feel like common-sense environmental laws.

Practically, these implications extend to education, cultural policy, and gender advocacy in Saudi Arabia and similar contexts. Educators and curriculum developers could incorporate critical readings of proverbs into language, literature, or civic education programs, encouraging students to interrogate how familiar sayings naturalize unequal distributions of domestic labor or reputational risk. Such initiatives might foster greater awareness of subtle ideological mechanisms in everyday speech, promoting more equitable gender discourse. For cultural institutions like the Saudi National Encyclopedia or heritage initiatives, the findings suggest value in expanding archival metadata to include critical annotations on gender and ecological dimensions, or in curating complementary collections that highlight contemporary reinterpretations by women speakers.

Recommendations for future research are both methodological and thematic. Ethnographically oriented studies could capture proverbs in vivo through interviews, focus groups, or recorded conversations, examining how Saudi women and men deploy, resist, or reinterpret these sayings in daily life particularly amid rapid social changes such as increased female workforce participation and evolving household dynamics. Digital ethnography would be especially fruitful, tracking proverb circulation on platforms like X (formerly Twitter), Instagram, or TikTok, where memes, reels, and threads often repurpose traditional sayings for humor, critique, or reinforcement. Comparative projects could extend the corpus across Arabian Peninsula regions (e.g., Emirati, Bahraini, or Yemeni proverbs) or contrast women-focused sayings with those centered on men, testing whether domestic-ecological motifs are distinctly gendered. Quantitative corpus-linguistic approaches might measure motif frequencies systematically, while interdisciplinary collaborations with anthropologists or sociologists could link proverbial patterns to measurable shifts in gender roles post-Vision 2030 reforms.

Ultimately, this study implies that engaging with vernacular forms like proverbs is essential for understanding how gender ideologies persist and adapt. By treating them as dynamic discourses

of domestic ecological governance rather than static folklore, scholars and practitioners can better address their role in both sustaining hierarchies and—potentially—opening spaces for contestation and change.

Declarations

Funding: This research received no specific grant from any funding agency in the public, commercial, or not-for-profit sectors.

Conflicts of Interest: The author declares no conflicts of interest.

Ethical Approval: Not applicable (as this is a textual analysis of publicly archived proverbs).

Data Availability: The proverb corpus is drawn from the Saudi National Encyclopedia (King Abdulaziz Public Library, 2007), publicly available online. No new data were generated.

Author Contributions: The author is solely responsible for conceptualization, methodology, analysis, and writing.

References

1. General Authority for Statistics (GASTAT). Labor Market Bulletin Q2 2025 [Internet]. Riyadh: General Authority for Statistics (GASTAT); 2025 [cited 2025 Dec 28]. Available from: https://www.stats.gov.sa/en/indicators/10

2. Saudi Arabia reports 67.1 percent labor force participation rate in Q2 2025. Economy Middle East [Internet]. 2025 Sept 30 [cited 2025 Dec 28]; Available from: https://economymiddleeast.com/news/saudi-arabia-reports-67-1-percent-labor-force-participation-rate-in-q2-2025/

3. Iovino S. Oppermann, S.(eds.), 2014, Material Ecocriticism. Bloomington-Indianapolis, Indiana University Press; 2014.

4. Oppermann S. Feminist ecocriticism: A posthumanist direction in ecocritical trajectory. In: International perspectives in feminist ecocriticism [Internet]. Routledge; 2013 [cited 2025 Nov 21]. p. 19–36. Available from: https://www.taylorfrancis.com/chapters/edit/10.4324/9780203520840-2/feminist-ecocriticism-serpil-oppermann

5. Plumwood V. Feminism and the Mastery of Nature [Internet]. Routledge; 2002 [cited 2025 Dec 22]. Available from:

https://www.taylorfrancis.com/books/mono/10.4324/9780203006757/feminism-mastery-nature-val-plumwood

6. Warren K. Ecofeminist philosophy: A western perspective on what it is and why it matters. Bloomsbury Publishing PLC; 2000.

7. Encyclopedia of the Kingdom of Saudi Arabia: Al Jawf Region [Internet]. Riyadh (Saudi Arabia): King Abdulaziz Public Library; 2007 [cited 2025 Dec 28]. First: some folk proverbs. Available from: https://saudiency.kapl.org.sa/Loader.aspx?pageid=38&BookID=148&PID=29

8. Alaimo S. Bodily natures: Science, environment, and the material self [Internet]. Indiana University Press; 2010 [cited 2025 Dec 22]. Available from: https://books.google.com/books?hl=en&lr=&id=xOw7_q-SZEcC&oi=fnd&pg=PP2&dq=Alaimo,+S.+(2010).+Bodily+natures:+Science,+environment,+and+the+material+self.+Bloomington,+IN:+Indiana+University+Press.&ots=BCmOaXpguV&sig=hRkQbB_9SOXPLPCbtsfYGnzH4S0

9. Mieder W. Proverbs: A handbook. 2004 [cited 2025 Dec 22]; Available from: https://www.academia.edu/download/52163076/wolfgang_mieder.pdf

10. Norrick NR. How proverbs mean: Semantic studies in English proverbs [Internet]. Vol. 27. Walter de Gruyter; 1985 [cited 2025 Dec 22]. Available from: https://books.google.com/books?hl=en&lr=&id=WtO1txl7DJwC&oi=fnd&pg=PA1&dq=Norrick,+N.+R.+(1985).+How+proverbs+mean:+Semantic+studies+in+English+proverbs.+Berlin:+Walter+de+Gruyter.&ots=0A1mKY1Qbb&sig=Cjz3p0v6rffMtW8ydNS1hRWtLBY

11. Lazar MM. Feminist Critical Discourse Analysis. In: Ehrlich S, Meyerhoff M, Holmes J, editors. The Handbook of Language, Gender, and Sexuality [Internet]. 1st edn Wiley; 2014 [cited 2025 Dec 8]. p. 180–99. Available from: https://onlinelibrary.wiley.com/doi/10.1002/9781118584248.ch9

12. Lazar MM. Feminist critical discourse analysis. In: The Routledge handbook of critical discourse studies [Internet]. Routledge; 2017 [cited 2025 Dec 8]. p. 372–87. Available from: https://www.taylorfrancis.com/chapters/edit/10.4324/9781315739342-26/feminist-critical-discourse-analysis-michelle-lazar

13. Ghafoori M, Elyas T. Power and Gender in Saudi Hijazi Proverbs: An Ideological-Cultural Study. J Psycholinguist Res. 2023 June;52(3):887–904.

14. Hassanein H, Mahzari M. A taxonomy of antonymy in Arabic: Egyptian and Saudi proverbs in comparison. Open Linguistics. 2021 June 7;7(1):200–22.

15. Honeck RP. A proverb in mind: The cognitive science of proverbial wit and wisdom [Internet]. Psychology Press; 2013 [cited 2025 Dec 22]. Available from: https://www.taylorfrancis.com/books/mono/10.4324/9780203771556/proverb-mind-richard-honeck

16. Al-Hussami AA, Al-Hiba M. Gender discrimination against women in Yemeni proverbs. Majallat Jamiʿat al-Mahrah lil-ʿUlum al-Insaniyyah (Al Mahrah University Journal for Humanities). 2023;4(2):461–82.

17. Al-Khawaldeh NN, Banikalef AA, Rababah LM, Khawaldeh AF. Ideological representations of women in Jordanian folk proverbs from the perspective of cultural semiotics. Humanities and Social Sciences Communications. 2024;11(1):1–10.

18. Leitner B, Hasani AS. Arabic Proverbs and Idioms as a Mirror of Gender and Society. Wiener Zeitschrift für die Kunde des Morgenlandes. 2021;111:99–120.

19. Balavi R, Nemati F, Alboghbeysh S. Representation of Women in the Arabic Proverbs of Khuzestan Natives: Applying Fairclough's Critical Discourse Analysis. Lisān-i Mubīn. 2022;

20. Fernandez LJT. Perpetuation of sexism through proverbs: The case of Martínez Kleiser's Refranero General Ideológico Español. Language & Communication. 2022;83:77–96.

21. Alasmari MA. Gender Representation in Southern Arabian Proverbs: A Critical Discourse Analysis. University of Tabuk Journal for Humanities and Social Sciences. 2024 Mar;4(1):228–51.

22. Bajubair A, Abdulla N. The Translatability of Hudhrami Women Proverbs into English among MA Translation Students at Al-Rayan University. Al-Andalus journal for Humanities & Social Sciences [Internet]. 2022 [cited 2025 Dec 22];9(55). Available from: https://aust.uni.ye/magazine/sh/2022/9/55/dda6314d42d6a1781ab72db609cce35b.pdf

23. Alharbi AS. Translating Saudi Najdi Dialect Proverbs into English: Challenges and Strategies for Preserving Cultural Meaning. International Journal of Linguistics, Literature and Translation. 2023;6(12):213–27.

24. Ismail SM. Translating dialect embedded in Najdi proverbs into English. Dialectologia: revista electrònica. 2017;93–107.

25. Alghamdi NA. Socio-pragmatic representation of animal in Al-Bahah proverbs: An ecolinguistic analysis. Utopía y Praxis Latinoamericana. 2019;24(6):274–84.

26. Newmark P. A textbook of translation [Internet]. Vol. 66. Prentice hall New York; 1988 [cited 2025 Dec 29]. Available from: https://www.academia.edu/download/45828515/A_Textbook_of_Translation_by_Peter_Newmark.pdf

27. Nida EA. Toward a science of translating: with special reference to principles and procedures involved in Bible translating [Internet]. Brill Archive; 1964 [cited 2025 Dec 29]. Available from: https://books.google.com/books?hl=en&lr=&id=YskUAAAAIAAJ&oi=fnd&pg=PP15&dq=28.+Nida+EA.+Toward+a+science+of+translating:+with+special+reference+to+principles+and+procedures+involved+in+Bible+translating.+Leiden:+Brill%3B+1964.&ots=trXER4dLLa&sig=gu4OJuZfKi9iiMyuGUTlyf7U6T0
