# Supplementary material for: Domestic ecologies of gender: Women, material life, and eco-folklore in Saudi proverbs
Source: PLoS One. 2026 Jun 10;21(6):e0348777. doi: 10.1371/journal.pone.0348777 (PMC13252737; doi:10.1371/journal.pone.0348777)
Supplement: Attachment — Submitted filename: Response to Reviewers.docx [file pone.0348777.s003.docx]

**Response to Reviewers**

I sincerely thank the reviewers for their careful reading of the manuscript and for their valuable comments. I greatly appreciate the positive evaluation of the study and have revised the manuscript where needed in response to the suggestions below.

**Reviewer #1**

**Comment:** Comprehensive, detailed analysis of paremiology concerning women from the theoretical framework of ecofeminism with positive results. The author presents a correct and self-explanatory abstract and keywords. Moreover, a sound introduction follows, together with the general and specific research objectives. An appropriate methodology is also presented.

**Response:** I sincerely appreciate this generous and encouraging assessment. Thank you for recognizing the clarity of the abstract, keywords, introduction, research objectives, and methodology. Your positive evaluation confirms the overall coherence and scholarly direction of the manuscript.

**Reviewer #2**

**Comment:** The manuscript is technically sound, and the data support the conclusions. The statistical analysis is not applicable in this research. The author has made all data underlying the findings in the manuscript fully available. The manuscript is presented in an intelligible fashion and written in standard English.

**Response:** Thank you very much for this positive and careful evaluation. I appreciate your recognition that the manuscript is technically sound, that the data support the conclusions, and that statistical analysis is not required for this type of research. I am also grateful for your acknowledgement of the clarity of presentation and the standard of English used in the manuscript.

**Reviewer #3**

**Comment:** No need to mention examples of proverbs in the introduction and the author can transform them to analysis section.

**Response:** Thank you for this helpful suggestion. I have revised the manuscript accordingly by removing the examples of proverbs from the introduction and relocating them to the analysis section. I believe this change improves the focus of the introduction and makes the analytical discussion more effective.

Please check the highlighted proverbs in the manuscript with the track changes on pp. 19, 21, 22, 23,and 24.

Once again, I sincerely thank the reviewers for their thoughtful comments and constructive feedback. Their observations have helped me improve the manuscript.
